# Peer Relationship Difficulties in Multiethnic Classrooms: A Longitudinal Study

**DOI:** 10.3390/bs15101430

**Published:** 2025-10-21

**Authors:** Maria Chiara Basilici, Federica Stefanelli, Annalaura Nocentini, Ersilia Menesini

**Affiliations:** Department of Education, Languages, Intercultures, Literatures and Psychology, University of Florence, Via di San Salvi, 12, Complesso di San Salvi Padiglione 26, 50135 Florence, Italy; mariachiara.basilici@unifi.it (M.C.B.); annalaura.nocentini@unifi.it (A.N.); ersilia.menesini@unifi.it (E.M.)

**Keywords:** peer relationship difficulties, immigrant background, multiethnic classrooms, classroom immigrant proportion, linear mixed model

## Abstract

Peer relationship difficulties during adolescence can significantly affect development. As classrooms become increasingly multiethnic, little is known about how native students navigate these contexts, while research on students with an immigrant background has mainly examined the onset of peer relationship difficulties. Moreover, the impact of the classroom ethnic composition—the proportion of students with an immigrant background relative to native students—remains unclear, with various theories offering conflicting perspectives. To address these gaps, this study examines the longitudinal development of peer relationship difficulties, considering students’ immigrant backgrounds and classroom ethnic composition. Two data collections were conducted (T1: December 2021/January 2022; T2: May/June 2022) in Italy. The sample included 604 first-year high school students (M_age_ = 15.16; SD_age_ = 0.56; 17.5% with an immigrant background; 52.9% males) nested within 30 classrooms across 8 schools. Results from the random intercept linear mixed model showed an increase in peer relationship difficulties for native students in medium and highly multiethnic classrooms, where the proportion of students with an immigrant background was above the sample mean, but not in low multiethnic classrooms (below the sample mean). Students with an immigrant background experienced an increase in peer relationship difficulties over time across all classroom conditions. Classroom ethnic composition plays a crucial role in shaping peer relationship difficulties, suggesting that the presence of multiple ethnic groups may present additional challenges. The study underscores the need for targeted, inclusive interventions and contributes to theoretical debates on the social dynamics of multiethnic classrooms.

## 1. Introduction

### 1.1. Peer Relationships in Adolescence

Peer relationships play a pivotal role in an individual’s development, particularly during adolescence, when peer groups are among the most significant and influential social contexts (e.g., Brown & Larson, 2009). Indeed, the development of positive peer relationships fosters various aspects of growth, including personality maturation (e.g., Hogan & Roberts, 2004), cognitive development (Parke et al., 2014), and social and emotional bonding (Swenson et al., 2008), as well as engagement in extracurricular and academic activities (Juvonen et al., 2012). Conversely, experiencing peer relationship difficulties (e.g., lack of friendship, peer rejection) can detrimentally impact self-esteem (Bishop & Inderbitzen, 1995) and is linked to psychopathological symptoms in adulthood (Bagwell et al., 2001). Therefore, peer relationships are crucially associated with overall well-being and mental health.

### 1.2. School in the European Context

One of the primary settings for social interactions and relationship development is the school, especially the classroom, which plays a key role. This is particularly marked in the European context, where students typically spend the entire school day in the same classroom with the same classmates. In recent years, the classroom ethnic composition has been significantly altered by increasing immigration trends in our country (IOM, 2022). Specifically, Italy has become the main entry point for migrants to Europe, with over 157,600 arrivals via the Mediterranean in 2023—the highest number since 2017. Of these, 26,800 were under the age of 18 (UNICEF, 2023). Consequently, classrooms are becoming increasingly diverse, bringing together native students with a growing number of students from immigrant backgrounds. In the 2021/2022 school year, 87,360 students in Italian schools (10.6% of the total) had an immigrant background, slightly up from 870,000 in 2015/2016. They come from nearly 200 countries, mainly Europe (44%), Africa (over 25%), Asia (20%), and Latin America (8%). Educational delay affects 25.4% of these students, compared to 8.1% of native students (Fondazione ISMU, 2023).

In this regard, it is essential to clarify how immigrant background is conceptualized and how ethnicity is defined. Ethnicity refers to a category based on common spoken language, religion, nationality, history, and other cultural factors that provide individuals with a sense of belonging to one group and distinction from others (Mishra, 2016). Being part of an ethnic group can shape individual culture, as ethnicity is a primary means through which culture is transmitted (Betancourt & López, 1993). However, the literature presents mixed methods and diverse operationalizations of ethnicity. In the European context, research typically focuses on immigrant background, operationalized through the country of birth or nationality of the student and their parents, which allows differentiation between first- and second-generation immigrants (e.g., Motti-Stefanidi et al., 2008; Palladino et al., 2020; Zaiceva & Zimmermann, 2008). By contrast, North American studies have traditionally relied on fixed ethno-racial categories and diversity indices, emphasizing race and genetically transmitted physical characteristics of human groups (e.g., Hanish & Guerra, 2000; S. Graham & Juvonen, 2002; Maynard et al., 2016; Mishra, 2016). Accordingly, whereas groups with an immigrant background are defined by their migration status (first- or second-generation), multiethnic groups comprise a wider diversity of ethnic backgrounds, encompassing both immigrant-origin individuals and native-born populations. This conceptual distinction is crucial for the European school context, where rapidly increasing demographic complexity necessitates clear, legally and socially salient criteria for studying social phenomena among students. Ultimately, this methodological divergence reflects the broader sociocultural and historical contexts of each region; for example, Europe’s long history of intra-regional migration makes social and legal dimensions more salient than racial categories in defining ingroups and outgroups (Basilici et al., 2024a).

### 1.3. Peer Relationships in Multiethnic Classrooms

Building relationships with peers who have different habits, customs, and practices poses a new challenge for students. Sociometric studies showed that students tend to prioritize relationships with peers who share their immigrant background, rather than with those from a different immigrant background (J. A. Graham & Cohen, 1997; Fortuin et al., 2014; Singleton & Asher, 1979). According to the theory of Ingroup Bias (Tajfel, 1982), this preference arises because individuals generally view members of their own group more positively, while finding it harder to relate to those from other groups. Consequently, native students, who typically make up the majority in the classrooms, are expected to encounter fewer peer relationship difficulties. However, as the classroom becomes more multiethnic—that is, including not only native students but also peers with diverse immigrant backgrounds—native students may have fewer opportunities to form relationships exclusively with other natives. These dynamics are further influenced by group hierarchies, which can complicate social interactions. Despite their importance, the experiences of native students in multiethnic settings remain understudied. For instance, a recent study indicates that exposure to different ethnic groups in adolescence may even heighten racial prejudice later in life (Goldman & Hopkins, 2020). However, there is a lack of research on the longitudinal development of peer relationship difficulties among native students in diverse classrooms.

Studies have primarily focused on the experiences of students with an immigrant background—referring to individuals whose immigrant background differs from that of the native population in the host country—highlighting that they often face peer relationship difficulties within multiethnic classroom. In fact, they are more likely to drop out of school due to issues with their peers (Bianchi et al., 2021; Makarova & Herzog, 2013). Such problems are frequently linked to the broader challenges of adapting to a new culture and custom, including learning a new language (J. W. Berry, 2009), which can hinder the ability to establish friendships (Sam & Berry, 2010). Additionally, students with an immigrant background, particularly first-generation immigrants, often experience rejection and isolation (Basilici et al., 2024a; Jansen et al., 2016; Plenty & Jonsson, 2017). However, multiethnic classrooms may mitigate these issues by increasing the likelihood that students with an immigrant background find peers with their same backgrounds (Boda et al., 2023).

### 1.4. The Role of Classroom Ethnic Composition

Following this reasoning, classroom ethnic composition may strongly influence peer relationships in school. However, the literature on this topic is mixed, and it remains unclear whether ethnically diverse classrooms alleviate or exacerbate peer relationship difficulties for both native students and those with an immigrant background.

On one hand, some studies suggest that greater ethnic diversity can reduce these difficulties, as daily interactions with classmates from different immigrant backgrounds may foster positive exchanges (Christ et al., 2022). For example, Bohman and Miklikowska (2021) using a five-year longitudinal panel of Swedish majority adolescents, found that classroom ethnic diversity indirectly reduced anti-immigrant attitudes through the formation of cross-ethnic friendships. Similarly, Lessard et al. (2019) conducted a study in California with students from diverse ethnic groups in multiethnic classrooms and confirmed that greater classroom diversity was associated with increased opportunities for cross-ethnic friendships. Finally, a longitudinal study involving Latino and African American students in 11 U.S. middle schools showed that higher classroom diversity was associated with reduced peer harassment (Juvonen et al., 2006). These findings align with Contact Theory (Allport, 1954), which posits that direct, positive relationships between students from different backgrounds are more likely to develop when certain conditions are met. The central mechanism is that contact, under specific optimal conditions, can disrupt negative stereotypes and reduce intergroup conflict. Four key conditions have been identified: equal status between groups, cooperation toward a common goal, and institutional support from authorities. In the school setting, fulfilling these conditions is essential for transforming multiethnic classrooms into environments that foster positive social outcomes. This framework highlights how both native and students with an immigrant background can benefit from opportunities to interact, socialize, and build lasting positive relationships.

On the other hand, some studies emphasize that multiethnic classrooms can exacerbate peer relationship difficulties. In such settings, students often form friendships primarily with peers from their own immigrant background rather than engaging in cross-ethnic relationships, particularly in schools where ethnic groups are of similar size and no clear majority exists (Goldsmith, 2004). Similarly, a study conducted in the Netherlands with 911 middle school students (Munniksma et al., 2017) found that greater classroom and neighborhood diversity were associated with stronger tendencies to form same-ethnic rather than cross-ethnic friendships, among both majority and minority students. The formation of distinct groups and a preference for one’s own group can lead to intolerant attitudes towards outgroups, potentially resulting in exclusionary or even aggression behaviors (Blalock, 1967), such as ethnic bullying (Bayram Özdemir et al., 2021), acculturative stress and related problems with peer engagement (Gándara & Contreras, 2009). This aligns with Intergroup Conflict Theory (J. C. Turner et al., 1979), which suggests that increased interactions among students from different ethnic groups can heighten conflict. It also corresponds to Social Dominance Theory (Sidanius et al., 1994), which posits that hierarchical structures in social systems, based on group distinctions, can give rise to conflict and oppression. In multiethnic classrooms, such hierarchies may intensify peer relationship difficulties, with students from dominant groups potentially exerting social control, while those from less dominant groups experience exclusion or marginalization.

More recently, the Power Imbalance Theory (S. Graham, 2006; Juvonen et al., 2006) has emphasized the role of the proportion of ethnic groups in classrooms. According to this theory, in ethnically diverse classrooms, students initially share a relatively balanced distribution of social power. However, as the degree of ethnically diverse groups increases, power imbalances tend to emerge. In such environments, minority groups often face disadvantages, with lower numerical representation, reduced social influence, and limited support compared to their native peers. This dynamic places them at a higher risk of experiencing peer relationship difficulties. Conversely, students from the majority ethnic group are more likely to encounter fewer peer relationship difficulties. On the other hand, when students with an immigrant background constitute the majority, they may have greater opportunities to connect with peers who share similar experiences, potentially reducing some peer relationship difficulties. Consistent with this, Bellmore et al. (2007) highlighted the key role of the classroom ethnic composition in peer relationships, finding that in settings with a clear numerical ethnic majority and minority, students in the minority group were less accepted and more frequently rejected by their majority peers. This pattern was attributed to both fewer chances for minority students to be nominated and the presence of in-group biases. In summary, these findings highlight that classroom ethnic composition is a crucial factor in understanding the development of peer relationship difficulties, as a student’s placement within a majority or minority group can shape social dynamics and interactions.

Notably, the operationalization of classroom ethnic composition differs across countries (Basilici et al., 2022). In Europe, it is typically measured as the percentage or proportion of students from different backgrounds within a classroom, whereas in North America, it is often assessed using diversity indices. These methodological differences influence how classroom diversity is assessed and interpreted, making it essential to consider the conceptualization of immigrant background when comparing findings.

Finally, since daily interactions with peers can shape relationship development, the analysis of peer relationship difficulties in multiethnic school classrooms should take a longitudinal approach. Previous research has highlighted the importance of examining relationships among peers from immigrant backgrounds over time during adolescence, as prolonged contact can lead to changes in interactions and attitudes (Titzmann et al., 2015; R. N. Turner & Feddes, 2011). However, to the best of our knowledge, no longitudinal studies to date have investigated peer relationship difficulties over time in such settings.

### 1.5. The Present Study

Overall, peer relationships are crucially associated with well-being and mental health (e.g., Parke et al., 2014; Bagwell et al., 2001). Despite that, literature on how students cope with peer relationships in multiethnic classrooms is still limited and inconclusive. Specifically, the perspective of native students is understudied, whereas research on students with an immigrant background mainly emphasized the emergence of peer relationship difficulties (e.g., Bianchi et al., 2021; Makarova & Herzog, 2013). Furthermore, the influence of the classroom ethnic composition in developing peer relationship difficulties remains ambiguous, with various theories offering conflicting perspectives (Allport, 1954; S. Graham, 2006; J. C. Turner et al., 1979). Finally, to date, no longitudinal studies have examined peer relationship difficulties over time in multiethnic classrooms. To fill these gaps, the aim of the present study is to analyze the development of peer relationship difficulties over time during the first year of high school, taking into account the potential moderating role of students’ immigrant background and of classroom immigrant proportion.

Given that individuals tend to favour relationships with in-group members over out-group members (Tajfel, 1982), and that the development of peer relationship difficulties is influenced by the power imbalance created by the classroom ethnic composition—where students belong to either the majority or the minority group (S. Graham, 2006; Juvonen et al., 2006)—we hypothesize that students are more likely to develop peer relationship difficulties in classrooms where there is a higher proportion of peers from different immigrant backgrounds and fewer peers from their own immigrant background. Specifically, considering a sample of students who are attending the same classroom together for the first time, we hypothesize that: (1) native students experience an increase in peer relationship difficulties over time in highly multiethnic classrooms, while (2) students with an immigrant background experience an increase in peer relationship difficulties over time in low multiethnic classrooms. The data for this study were obtained exclusively from the control group of a broader intervention trial implemented in schools.

## 2. Materials and Methods

### 2.1. Participants

Overall, 667 first-year high school students (i.e., grade 9) participated in the current study. However, 3 students were removed from the sample as outliers due to their age^1^. Therefore, the final sample included 664 students (M_age_ = 15.16; SD_age_ = 0.56; Min_age_ = 14; Max_age_ = 19)^2^ nested in 30 classrooms from 8 schools (i.e., 50% technical or vocational and, 50% lyceum). Of these, 42.2% (*n* = 280) were female, 52.9% (*n* = 351) were male, and 5% (*n* = 33) stated that they preferred not to answer the question.

Students’ immigrant background was defined based on their parents’ country of birth, following a common operationalization used in Europe as a proxy for ethnicity (Basilici et al., 2022). This approach is particularly relevant in the Italian context: citizenship status is primarily determined by the principle of ius sanguinis (Italy L. No. 91, 1992), requiring Italian descent as a condition for acquisition. Consequently, parental country of birth directly determined a students’ legal status and institutional categorization, making it a highly salient measure. Following this framework, students with at least one Italian parent were classified as natives, while students with both parents born abroad (i.e., first generation immigrants) and students born in Italy to foreign-born parents (i.e., second generation immigrants) were classified as students with an immigrant background. Accordingly, 81.9% (*n* = 544) were natives and 17.5% (*n* = 116) were students with an immigrant background. Unfortunately, the nationality of 0.6% (*n* = 4) of the students could not be determined due to missing data.

Students reported the following countries of birth: Albania (1.5%), Bangladesh (0.3%), Chile (0.2%), China (0.6%), Cuba (0.2%), Ethiopia (0.2%), Philippines (0.2%), Greece (0.2%), Honduras (0.2%), India (0.2%), Italy (94.3%) England (0.2%), Morocco (0.2%), Netherlands (0.2%), Pakistan (0.3%), Peru (0.2%), Dominican Republic (0.2%), Romania (0.3%), Russia (0.2%), Spain (0.2%), United States (0.2%), Switzerland (0.2%), Thailand (0.2%). The mothers’ countries of birth included: Africa (not specified country; 0.2%), Albania (6.0%), Bangladesh (0.2%), Brazil (0.3%), Canada (0.2%), China (4.5%), Colombia (0.2%), Congo (0.2%), Cuba (0.2%), Philippines (0.9%), France (0.2%), Germany (0.2%), Japan (0.3%), Honduras (0.2%), India (0.2%), England (0.3%), Turkey (0.2%), Italy (77.7%), Morocco (2%), Moldova (0.3%), Nigeria (0.3%), Pakistan (0.3%), Peru (0.2%), Poland (0.5%), Dominican Republic (0.3%), Romania (1.1%), Russia (0.5%), Scotland (0.2%), Slovenia (0.2%), Spain (0.3%), Sri Lanka (0.2%), Switzerland (0.2%), Thailand (0.2%), Tunisia (0.6%), Ukraine (0.5%), United States (0.2%). The fathers’ countries of birth included: Africa (not specified country; 0.5%), Albania (6.5%), America (0.3%), Bangladesh (0.3%), Brazil (0.3%), China (4.5%), Denmark (0.2%), Philippines (0.8%), France (0.2%), Greece (0.2%), Honduras (0.2%), England (0.2%), Italy (79.2%), Morocco (2.3%), Moldova (0.3%), Nigeria (0.3%), Norway (0.2%), Netherlands (0.2%), Pakistan (0.3%), Peru (0.2%), Poland (0.2%), Dominican Republic (0.2%), Romania (0.9%), Senegal (0.2%), Spain (0.5%), Sri Lanka (0.2%), Switzerland (0.2%), Tunisia (0.7%).

### 2.2. Procedures

As part of the National PRIN project 2017 “Prejudicial bullying involving ethnic groups: Understanding mechanisms and translating knowledge into effective interventions”, participants in this study served as a control group for the evaluation of an intervention aimed at preventing ethnic bullying in schools. Recruitment was based on a voluntary census, following a call for participation extended to all high schools in seven provinces in Tuscany region: Livorno, Lucca, Massa Carrara, Firenze, Pisa, Pistoia, Prato. The experimental procedures adhered to Helsinki Declaration of 1964 and were also approved by the Ethic Committee of the University of Florence.

Two waves of data collection were conducted: the first in December 2021/January 2022, approximately three months after the beginning of the school year, when students had been placed in the same classroom for the first time; and the second in May/June 2022, at the end of the school year, following consistent daily contact throughout the year.

Students completed the questionnaire during school hours, under the supervision of their teachers and trained researchers. Participants were assured that their responses would remain anonymous. Each school principal approved the agreement, and each student completed a consent form.

### 2.3. Measures

Peer relationship difficulties were measured using the Peer Relationship Problems (PRP) subscale of the Strengths and Difficulties Questionnaire (SDQ; Goodman, 1997), which assesses levels of involvement in age-appropriate peer relationships (i.e., “I am usually on my own. I generally play alone or keep to myself”; “I have one good friend or more” (reversed item); “Other people of my age generally like me” (reversed item)”; “Other young people pick me or bully me”; “I get on better with adults than with people my own age”. Items were rated on a three-point Likert (0 = not true; 1 = somewhat true; 2 = certainly true). The scale showed good internal consistency at each data collection point (i.e., McDonald’s ω is 0.70 a T1 and 0.69 at T2).

Classroom Immigrant Proportion (CIP) was defined by the proportion of natives and students with an immigrant background in each classroom. It should be noted that this measure does not account for the representation of specific ethnic groups within the immigrant student population. This methodological approach is supported by socio-legal dimensions which form the main basis for distinguishing native students from those with an immigrant background in the Italian context. The proportion ranged from 0.00 in the classroom groups with no students with an immigrant background to 60.00 in the more multiethnic classroom, with an average of 17.76 (*SD* = 16.75). Notably, this data is roughly in line with the national data, where approximately 20% of students with an immigrant background attend high school (e.g., 23.3% in 2019/2020; 25.2% in 2020/2021; Ministry of Education, 2021). Descriptive statistics of CIP are reported in Table 1.

### 2.4. Data Analysis

First, the retention rate and Little’s (1988) Missing Completely at Random (MCAR) test were analyzed to determine whether all participants could be included in the study. Second, continuous variables were centered to reduce multicollinearity (Shieh, 2011). Third, the distribution of the outcome variable (i.e., PRP) was examined to assess symmetry, and it was found to be normally distributed (*A* = 0.761; *K* = 0.171; Wah, 2025). Finally, because the measurement occasions were within individuals (i.e., student identification number at T1 and T2), and participants were nested within classrooms and schools, preliminary analysis were conducted to examine the potential effects of these clustering variables on the variance of PRP.

We used a random intercept linear mixed model (Singmann & Kellen, 2019) with PRP as the outcome variable and student identification, classrooms, and schools as cluster effects to determine whether the three cluster effects contributed to the variance in PRP. Mixed methods are appropriate for addressing complex situations in which participants are nested within a hierarchical structure. There was sufficient within-student variability to justify consideration of student-level (ICC = 0.616), classroom-level (ICC = 0.110), and school-level (ICC = 0.133) effects on PRP in a mixed model. Therefore, cluster effects were included in the analysis. Indeed, an ICC of 0.05 is generally required to justify the inclusion of a cluster variable in a multilevel analysis (Heck et al., 2013).

Then, a series of random intercept linear mixed models with full-information maximum likelihood (ML) estimation were run and compared, considering PRP as the outcome variable, and student identification, classrooms and schools as cluster variables. Specifically, at each step, we compared a simpler model with a more complex one to verify that the hypothesized model (from now, Model 3) was also the best model in terms of both fit and parsimony. If the predictive power of the two models is equivalent, the simpler model (the one with fewer predictors) is preferred. Specifically, we tested and compared the following four models: (1) Model 0, without predictors of PRP; (2) Model 1, including only TIME as a predictor of PRP; (2) Model 2, including the interaction term TIME × EC to explore whether the classroom immigrant proportion moderated the relationship between TIME and PRP; (4) Model 3, including the interaction term TIME × CIP × IMMIGRANT BACKGROUND to test whether changes in PRP were moderated by the interaction between TIME, CIP, and IMMIGRANT BACKGROUND. The triple interaction of Model 3 means that the independent variable (TIME), and the two moderators (CIP and IMMIGRANT BACKGROUND), interact in their totality by producing several different effects on the dependent variable (PRP).

To compare the four models of PRP and select the most statistically supported one, we used Akaike weights, which range from 0 to 1, and represent the probability that a model will predict new data (Wagenmakers & Farrell, 2004). We also reported each model’s explained variance for descriptive reasons, as condition R^2^ is appropriate for linear mixed models. The model with the highest Akaike weight was selected, unless the improvement in predictive performance was negligible, in which case the simpler model was retained to preserve parsimony. To perform the mixed models, we used the GAMLj module (Gallucci, 2020) in Jamovi (Version 2.3.21.0).

## 3. Results

Retention rates between the two consecutive assessments ranged from 88.70% to 77.56. Missing Completely at Random (MCAR) test yielded a non-significant result (χ^2^(2) = 4.579, *p* = 0.101), indicating that the null hypothesis that the data were missing completely at random could not be rejected. Therefore, all participants with at least one available time point were included in the analysis.

Descriptive statistics and bivariate associations between continuous variables are reported in Table 2.

Results of the models’ comparison are reported in Table 3.

As shown in Table 3, Akaike weights provided support for Model 3, the model including the three-way interaction Time × CIP × IMMIGRANT BACKGROUND as a predictor of PRP. Specifically, Model 3 (i.e., the best model) contained 98% of the total explanation that can be found in the full set of assessed models. The parameters of Model 3 are reported in Table 4.

As shown in Table 4, the two-way interactions included in the best model—TIME × IMMIGRANT BACKGROUND, TIME × CIP, and CIP × IMMIGRANT BACKGROUND—were not significant (*β* = −0.0972, *p* = 0.067; *β* = 0.0003, *p* = 0.768; *β* = 0.0013, *p* = 0.542, respectively). This suggests that the effect of time on peer relationship difficulties does not vary based on students’ immigrant background or the classroom immigrant proportion. Similarly, the effect of classroom immigrant proportion on peer relationship difficulties does not vary based on students’ immigrant background.

While the three-way interaction included in the best model was significant with b = 0.007, *SE* = 0.003, *p* = 0.001. Thus, Model 3 suggested that the level of CIP moderated the relationship between TIME and PRP, conditional on IMMIGRANT BACKGROUND.

**Figure 1:**
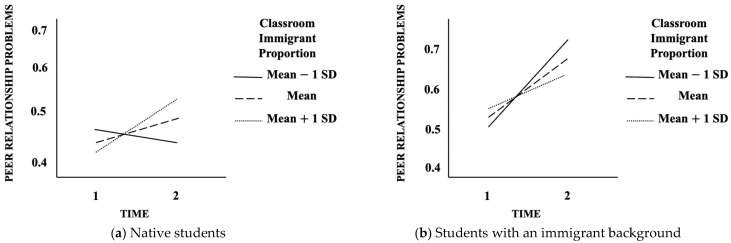
Graphical representations of estimated parameters derived from Model 3 split by IMMIGRANT BACKGROUND: (**a**) natives’ results; (**b**) students with an immigrant background’s results.

Analysis of the simple effects of the interaction TIME × CIP × IMMIGRANT BACKGROUND showed (Figure 1a) that native students in the low multiethnic classrooms experienced no change in PRP over time (CIP − 1 *SD* below the mean; *b* = −0.0236; *p* = 0.288). Instead, there was a significant increase in PRP over time in average multiethnic classrooms (*b* = 0.0463; *p* = 0.006) and highly multiethnic classrooms (CIP +1 *SD* above the mean; *b* = 0.1161; *p <* 0.001). For example, moving from Time 1 to Time 2, a native student in a low multiethnic classroom (–1 *SD*) showed a non-significant decrease of 0.024 standard deviations in peer relationship problems. By contrast, in average multiethnic classrooms the increase was about 0.046 standard deviations, while in highly multiethnic classrooms (+1 *SD*) it reached 0.116 standard deviations.

For students with an immigrant background (Figure 1b) PRP significantly increased over time in the low multiethnic classrooms (CIP − 1 *SD* below the mean; *b* = 0.2018; *p* = 0.008), average multiethnic classrooms (*b* = 0.1435; *p* = 0.005) and highly multiethnic classrooms (CIP +1 *SD* above the mean; *b* = 0.0852; *p* = 0.028). For example, moving from Time 1 to Time 2, peer relationship problems increased by about 0.201 standard deviations in low multiethnic classrooms, by 0.143 standard deviations in average multiethnic classrooms, and by 0.085 standard deviations in highly multiethnic classrooms.

## 4. Discussion

The aim of the present study was to analyze the development of peer relationship problems over time during the first year of high school, taking into account the potential moderating role of students’ immigrant background and of classroom immigrant proportion. This topic is particularly relevant in adolescence, a developmental period when peer groups represent one of the most significant and influential social contexts (e.g., Brown & Larson, 2009), and the quality of peer relationships can have long-lasting consequences that extend well into adulthood (Bagwell et al., 2001).

Our findings partially support the hypotheses, showing that peer relationship difficulties increased among native students in medium and highly multiethnic classrooms, and among students with an immigrant background regardless of the classroom’s ethnic composition. Results are coherent with existing research and theoretical frameworks in several directions. The increase in peer relationship difficulties in medium and highly multiethnic classrooms for native students suggests that such environments may intensify interpersonal conflicts and challenges, in line with the Intergroup Conflict Theory (J. C. Turner et al., 1979) and the Social Dominance Theory (Sidanius et al., 1994). Indeed, increased interactions between different ethnic groups may have heightened tensions and conflict, resulting in a rise in overall peer relationship difficulties. On the contrary, the lack of significant changes in peer relationship difficulties in low multiethnic classrooms highlight native students’ tendency to develop relationships with peers who share their same immigrant background rather than with those from a different one, as observed in Ingroup Bias (Tajfel, 1982) and previous sociometric studies (J. A. Graham & Cohen, 1997; Singleton & Asher, 1979). Lastly, these results align with Power Imbalance Theory (S. Graham, 2006; Juvonen et al., 2006). Indeed, in low multiethnic classrooms, in which there were not significant changes in problematic peer difficulties, native students were the dominant group. However, as the number of students with an immigrant background increased in medium and highly multiethnic classrooms, the numerical dominance of native students likely diminished, resulting in a decrease in their social influence within the group. This shift in power dynamics may have contributed to a higher likelihood of peer relationship difficulties, as the diminished majority status of native students could have disrupted the existing social hierarchy. It should be noted that the first assessment (T1) occurred after only three months, which may be a relatively short period for full power dynamics to develop. Nevertheless, the observed patterns suggest emerging trends in peer relationship difficulties among native students in classrooms with higher proportions of students with an immigrant background.

Findings regarding students with an immigrant background only partially support our hypothesis. The results showed an increase in peer relationship difficulties over time across all conditions examined, namely, low, medium, and highly multiethnic classrooms. This is consistent with previous research showing that students with an immigrant background often face peer-related challenges that can lead to difficulties in forming friendships (Sam & Berry, 2010) and in dropping out of school (Bianchi et al., 2021; Makarova & Herzog, 2013). In the present study, we did not expect an increase in peer relationship difficulties for students with an immigrant background in highly multiethnic classrooms, because, following the Ingroup Bias (Tajfel, 1982), they had more opportunities to interact with peers who shared their same immigrant background. However, this may not have been the case, as these students likely remained a numerical minority within the classroom. An increase in the proportion of students with immigrant background may exacerbate power imbalances (S. Graham, 2006; Juvonen et al., 2006), leading to heightened tensions and conflicts between groups, as suggested by the Intergroup Conflict Theory (J. C. Turner et al., 1979), and ultimately to more peer relationship difficulties. Notably, this study focused specifically on classroom immigrant proportion—that is, the ratio of native students to students with an immigrant background—without considering the representation of specific ethnic groups. In the present study, this was not feasible due to theoretical considerations and the sociopolitical context in which the data were collected, which did not allow for a meaningful classification of students into smaller ethnic groups. However, to gain a more comprehensive understanding of these findings, it would be beneficial for future studies to adopt alternative methods for analyzing ethnic groups within classrooms (e.g., Basilici et al., 2022), making use of indices that measure the concentration of each ethnic group, such as the Simpson Diversity Index (Simpson, 1949) or the Herfindahl Index (Putnam, 2007).

Furthermore, our findings diverge from some previous research (Bohman & Miklikowska, 2021; Christ et al., 2022; Lessard et al., 2019) and from the Contact Theory (Allport, 1954), which posits that interethnic contact fosters positive and cooperative interactions. In contrast, this study revealed that native students experienced an increase in peer relationship difficulties over time in medium and highly multiethnic classrooms, while students with an immigrant background faced these challenges across all classroom conditions (i.e., low, medium and highly multiethnic classrooms). These results are not surprising in the European context (Basilici et al., 2022), particularly in Italy (Basilici et al., 2024a), where multiethnic classrooms are of recent origin and at increased risk for intergroup conflict and ethnic bullying. This situation may be further influenced by recent political developments in Europe, where migration, especially the irregular type, has become a prominent issue (IOM, 2022). According to an ecological approach (Bronfenbrenner, 1979), these political debates and conflicts at a macrosocial level could escalate tensions among students from different immigrant backgrounds, even within microsystems like school environments and classrooms. Native students may engage in discriminatory behaviors and exclude those with a different immigrant background, while perceived rejection may lead students with an immigrant background to adopt separation as an acculturation strategy, thereby avoiding contact with other cultures (J. W. Berry, 2009). Together, these dynamics could contribute to an increase in peer relationship difficulties for both natives and students with an immigrant background, especially in multiethnic contexts. An additional explanation may be that interethnic contact is not sufficient to reduce prejudice or foster positive peer relationships. According to Allport’s (1954) Contact Theory, such outcomes are more likely when specific conditions are met: equal status among peers, the pursuit of common goals, cooperation, and support from relevant authorities. In school contexts, however, these conditions are not always guaranteed. For example, multiethnic classrooms may struggle to provide equal status and cooperative learning opportunities, while teachers often lack adequate training or institutional support to manage increasingly diverse groups. Our findings may therefore reflect not only the challenges of diversity but also the absence of the optimal conditions required for intergroup contact to promote positive and inclusive relationships (e.g., Zambuto et al., 2022). Notably, this study relies on a two-wave longitudinal design, which should be considered when interpreting the results; future studies could replicate the research with additional waves to better capture changes and developments over time.

Overall, these findings highlight significant challenges in multiethnic classrooms, where belonging to either the in-group or out-group plays a crucial role in shaping peer relationships. While these results may seem discouraging, group membership can also be influenced by factors such as language or cultural barriers (e.g., J. W. Berry, 2009), which were not analyzed in this paper. Moreover, the crucial role of in-group and out-group dynamics could suggest the existence of a threshold in classroom immigrant proportion—one not yet explored in the literature—beyond which distinctions between groups become less pronounced. However, if this threshold is exceeded, group divisions may intensify, potentially exacerbating intergroup conflict and hindering peer relationships. Future research could further explore this aspect.

From a practical standpoint, the present study underscores the need for interventions and educational policies —such as peer education programs, cooperative learning activities, and peer mentoring—that promote cooperation, inclusion, and the development of positive peer relationships among all students, regardless of their immigrant backgrounds. Without such interventions, peer relationship difficulties may create a negative environment affecting both natives and students from an immigrant background. In our increasingly diverse and globalizing world, a key challenge is to engage effectively and respectfully in intercultural interactions and dialogues with peers with different immigrant background, thereby enhancing the well-being of both individuals and society as a whole (J. Berry, 2016).

Several shortcomings limit the interpretability of the current findings. First, examining classroom immigrant proportion limits the depth of our understanding of the topic. Future research conducted in contexts where ethnic diversity can be operationalized using diversity indices should replicate this analysis, assessing classroom ethnic diversity more comprehensively, including the relative representation of each ethnic group within the classroom. Second, the research only focused on Italian schools, which may restrict the generalizability of the results. Third, it did not consider other age groups, indicating that future research should include participants from different ages and cultural contexts. Then, our analysis overlooked potential covariates that might influence the development of peer relationship difficulties in schools. Bellmore et al. (2007) suggest that in more multiethnic classrooms with a greater number of ethnic groups, acceptance, rejection, and social dynamics among students are also influenced by individual behaviors, social–cognitive skills, and personality traits. It would also be important to consider participants’ socioeconomic status to better understand their social context, as well as language proficiency, which may pose an additional barrier to integration and influence peer relationships (e.g., J. Berry, 2016). Furthermore, this study relies on only two time points. While this longitudinal design allows us to examine changes over time, the use of only two measurement waves limits the possibility of capturing the shape and complexity of developmental trajectories. Future research with more extended longitudinal designs and multiple measurement waves would allow a more fine-grained understanding of how these processes unfold over time and whether different subgroups of adolescents follow distinct developmental pathways. Again, the self-report nature of the measures may have increased the possibility of inflated associations due to potential social desirability bias. Finally, it is noteworthy that the data collection occurred in 2022, when Italian students were just back in school after a long period of closures and limitations due to the COVID-19 pandemic. Restrictions and rules to face with pandemic were still active in that period, although mitigated and scholars cautioned against returning students to school (Basilici et al., 2023), as this macrosocial event had a major impact on adolescents’ mental health and social interactions (e.g., Branje & Morris, 2021). Therefore, it’s possible that other unconsidered risk factors influenced the results of the present study.

Overall, despite these limitations, this study represents the first longitudinal study analyzing peer relationship difficulties in schools from the perspectives of both native students and those with an immigrant background, while also considering the role of classroom immigrant proportion. As also highlighted by the R-squared value, the findings underscore the importance of examining social behaviors, such as peer relationship problems, through both within-group and between-group approaches, and highlight the need to analyze the interactions between personal and structural factors. Finally, this study contributes valuable insights to the literature on interethnic peer relationships and offers some relevant suggestions for both researchers and practitioners.

## Figures and Tables

**Table 1:** Descriptive statistics of Classroom Immigrant Proportion.

Classroom	Classroom Immigrant Proportion
1	0.0
2	0.0
3	3.57
4	3.85
5	4.17
6	4.35
7	4.35
8	4.55
9	7.41
10	7.69
11	8.00
12	8.00
13	9.52
14	10.53
15	11.11
16	12.50
17	12.50
18	15.79
19	20.69
20	21.43
21	21.74
22	23.53
23	30
24	33.33
25	38.10
26	40
27	43.48
28	45.45
29	56.52
30	60

**Table 2:** Descriptive statistics of continuous variables: Mean, associated standard deviations, and Pearsons’s bivariate correlations.

	N	Mean (*SD*)	1.	2.	3.
1. T1 PRP	589	0.46 (0.39)	--	--	--
2. T2 PRP	515	0.49 (0.40)	0.640 **	--	--
3. CIP	664	17.76 (16.75)	0.048	0.136 **	--
4. IMMIGRANT BACKGROUND	660	--	--	--	--

Note. N = Sample size; ** *p* < 0.001; *SD* = Standard Deviation.

**Table 3:** Akaike weights and conditional R^2^ for compared models on PRP.

	Akaike Weights	R^2^
Model 0	<0.001	0.641
Model 1, Time	<0.001	0.645
Model 2, Time × CIP	0.002	0.649
Model 3, Time × CIP × IMMIGRANT BACKGROUND	0.989	0.660

**Table 4:** Mixed model predicting PRP.

Effect	Estimate	SE	*p* Value
Fixed effects			
Intercept	0.5448	0.042	<0.001
Time	0.9488	0.026	<0.001
CIP	−0.0001	0.001	0.947
IMMIGRANT BACKGROUND	−0.1268	0.047	0.007
TIME × IMMIGRANT BACKGROUND	−0.0972	0.053	0.067
TIME × CIP	0.0003	0.001	0.768
CIP × IMMIGRANT BACKGROUND	0.0013	0.002	0.542
TIME × EC × IMMIGRANT BACKGROUND	0.0077	0.003	0.001

Note. Number of observations = 1098; Students’ identification = 660; Classrooms = 30; Schools = 8.

## Data Availability

The data that support the findings of this study are available on request from the corresponding author.

## Notes

1 The three students who claimed to be 20, 21, and 23 years old were found to be ineligible for the class in which they were enrolled. Therefore, their data was ignored for lack of validity.

2 Of all students, 3.3% were 14 years old, 81.8% were 15 years old, 11.7% were 16 years old, 2.3% were 17 years old, 0.8% were 18 years old, and 0.2% were 19 years old.

## Abbreviations

The following abbreviations are used in this manuscript:

PRP	Peer Relationship Problems
CIP	Classroom Immigrant Proportion
